# An evolutionarily conserved interaction of tumor suppressor protein Pdcd4 with the poly(A)-binding protein contributes to translation suppression by Pdcd4

**DOI:** 10.1093/nar/gku800

**Published:** 2014-09-04

**Authors:** Olesja Fehler, Priyanka Singh, Astrid Haas, Diana Ulrich, Jan P. Müller, Johanna Ohnheiser, Karl-Heinz Klempnauer

**Affiliations:** Institute for Biochemistry, Westfälische-Wilhelms-Universität Münster, Wilhelm-Klemm-Str. 2, D-48149 Münster, Germany

## Abstract

The tumor suppressor protein programmed cell death 4 (Pdcd4) has been implicated in the translational regulation of specific mRNAs, however, the identities of the natural Pdcd4 target mRNAs and the mechanisms by which Pdcd4 affects their translation are not well understood. Pdcd4 binds to the eukaryotic translation initiation factor eIF4A and inhibits its helicase activity, which has suggested that Pdcd4 suppresses translation initiation of mRNAs containing structured 5′-untranslated regions. Recent work has revealed a second inhibitory mechanism, which is eIF4A-independent and involves direct RNA-binding of Pdcd4 to the target mRNAs. We have now identified the poly(A)-binding protein (PABP) as a novel direct interaction partner of Pdcd4. The ability to interact with PABP is shared between human and Drosophila Pdcd4, indicating that it has been highly conserved during evolution. Mutants of Pdcd4 that have lost the ability to interact with PABP fail to stably associate with ribosomal complexes in sucrose density gradients and to suppress translation, as exemplified by c-*myb* mRNA. Overall, our work identifies PABP as a novel functionally relevant Pdcd4 interaction partner that contributes to the regulation of translation by Pdcd4.

## INTRODUCTION

The tumor suppressor gene *Pdcd4* (*programmed cell death 4*) has been implicated in the development and progression of a multitude of human tumors, including cancer of the lung, colon, liver and breast and glioblastoma (1–6). The expression of Pdcd4 is frequently downregulated in these tumors, often in conjunction with increased expression of oncogenic micro-RNA miR-21, which targets the 3′ untranslated region (UTR) of *Pdcd4* mRNA (7–9). Downregulation of Pdcd4 protein expression appears to contribute to tumor development in different ways: several studies have shown that decreased Pdcd4 expression increases the mobility and invasiveness of tumor cells (5,6,9–11). In addition, decreased Pdcd4 expression appears to modulate the cellular response to DNA damage (12–15).

Pdcd4 is a highly conserved phosphoprotein that is able to shuttle between nucleus and cytoplasm (16,17). The subcellular localization of Pdcd4 is controlled by protein kinase Akt-mediated phosphorylation (17). Pdcd4 contains two so-called MA-3 domains, which are located in the central and the C-terminal parts of the protein, and an N-terminal domain involved in RNA binding. The MA-3 domains serve as binding regions for the translation initiation factor eIF4A (18–24), whereas the N-terminal domain interacts with specific RNA secondary structures as well as with certain proteins, such as the scaffold protein Daxx and the protein arginine methyltransferase 5 (15,16,25–28). Several studies have shown that Pdcd4 affects the activities of several transcription factors, such as c-Jun (29,30), Sp1 (11), Twist1 (31), p53 (12,15) and NF-kB (32) and thereby controls the transcription of specific genes. In addition, Pdcd4 acts as a translation suppressor of specific mRNAs. The interaction of Pdcd4 with eIF4A inhibits the RNA helicase activity of eIF4A, which is required to unwind secondary structures in 5′ UTRs of mRNAs (18,33). It is therefore thought that Pdcd4 suppresses the translation initiation of mRNAs with structured 5′ UTRs. This has been confirmed by using artificial RNAs containing stable hairpin structures in their 5′ UTRs (18,33) and, more recently, by the identification of p53 mRNA as a physiological Pdcd4 target mRNA with a highly structured 5′ UTR (14). We have recently shown that Pdcd4 also suppresses the translation of c-*myb* and A-*myb* mRNAs (27,28). The inhibitory mechanism in these cases appears not to involve the interaction with eIF4A but to depend on the binding of Pdcd4 to secondary structure motifs located in the coding regions of these RNAs. Furthermore, this work has suggested that Pdcd4 inhibits translation elongation rather than translation initiation in these cases (28).

We have now examined the role of Pdcd4 as a translation suppressor in more detail and identified the poly(A)-binding protein (PABP) as a novel direct interaction partner of Pdcd4. Our work shows that this interaction has been conserved during evolution and suggests that it plays an important role in tethering Pdcd4 to target RNAs.

## MATERIALS AND METHODS

### Eukaryotic expression vectors

pCDNA3-chc-Myb is an expression vector for full-length chicken c-Myb (27). The expression vector for wild-type human Pdcd4 has been described in (25). pCDNA4-hPdcd4-V123A/W124A and pCDNA4-hPdcd4-K114A/K115A encode mutant human Pdcd4 proteins containing the indicated amino acid substitutions in the N-terminal part of Pdcd4. pCDNA4-hPdcd4-mut4 encodes a Pdcd4 mutant in which E249, D253, D414 and D418 were replaced by alanine (14). pEGFP-eIF4G-Nt encodes a fusion protein of EGFP and amino acids 84–224 of human eIF4G and was generated by subcloning the corresponding part of the eIF4G coding region into plasmid pEGFP-C1. The expression vector for Myc-tagged human PABP was obtained from H. Okano (34). pCDNA3–6xMyc-PABP-RRM1+2, pCDNA3–6xMyc-PABP-RRM3+4 and pCDNA3–6xMyc-PABP-Ct are expression vectors for Myc-tagged subregions of human PABP. An expression vector for Flag-tagged Tiar was obtained from C. Gueydan.

Transient transfection of QT6 fibroblasts was performed by Calcium-phosphate coprecipitation as described in (30) or by lipofectamine, according to the manufacturer's protocol. The β-galactosidase plasmid CMV-β (Invitrogen) was included to monitor and normalize the transfection efficiencies.

### Bacterial expression vectors

pGex-6P2-hPdcd4wt encodes full-length human Pdcd4 fused to GST (25). pGex-6P2-hPdcd4-RBDstop is a derivative generated by introducing a translation stop codon at amino acid position 155 of Pdcd4. pGex-6P2-hPdcd4-mut4 encodes a GST-Pdcd4 fusion protein in which Glu249, Asp253, Asp414 and Asp418 were mutated to Ala. pGex-6P2-hPdcd4-mut1, pGex-6P2-hPdcd4-mut2 and pGex-6P2-hPdcd4-DM encode GST-Pdcd4 fusion proteins carrying amino acid substitutions in the RNA-binding domain that disrupt the RNA-binding activity (25). pGex-6P2-hPdcd4-R110A, pGex-6P2-hPdcd4-R110K, pGex-6P2-hPdcd4-K114A/K115A, pGex-6P2-hPdcd4-K121A, pGex-6P2-hPdcd4-V123A/W124A and pGex-6P2-hPdcd4-A118E encode GST-Pdcd4 fusion proteins carrying the indicated amino acid substitutions. pGex-6P2-ΔRBD encodes a GST-Pdcd4 fusion protein with an internal deletion of amino acids 66–150 of Pdcd4. pGex-6P2-DmPdcd4 encodes a *Drosophila melanogaster* Pdcd4 GST fusion protein and was generated by amplifying the coding region of Drosophila CG10990 with the primers 5′-CGTGGAATTCCCATGGAAGTGGAATCGAAT-3′ (forward) and 5′-ATATGCGGCCGCTTAATCACGCATGGT-3′ (reverse) and cloning it between the EcoRI and NotI sites of pGex-6P2. pGEX-4T1-DmPdcd4-RBDstop encodes a related fusion protein containing only the N-terminal domain of DmPdcd4. To construct this plasmid the corresponding coding region was amplified using the primers 5′-GGCGAATTCATGGAAGTGGAATCGAAT-3′ and 5′-AGGCTCGAGTTACAGCTCCACATTACGATC-3′ and cloning it between the EcoRI and XhoI sites of pGex-4T1. The bacterial expression vector for mouse His-PABP-RRM1–4 (amino acids 1–375) was a gift from Lorna Waters (Department of Biochemistry, Leicester, UK).

### Antibodies

c-Myb was detected with Myb-specific monoclonal antibodies 5E11 (35). Pdcd4 was detected with a rabbit antiserum against the N-terminus of human Pdcd4 (12). Antibodies against GFP (Roche Diagnostics, Mannheim, Germany), eIF4A, eIF4G, eIF4E, PABP (Cell Signalling Technology), Y14 (Santa Cruz Biotechnology) and the Flag, Myc and His-tag were obtained from commercial suppliers. Antiserum against Drosophila PABP was kindly provided by E. Izaurralde (Tübingen, Germany).

### GFP-Trap and co-immunoprecipitation

QT6 fibroblasts transfected with the desired expression vectors were lysed 24 h after transfection in egg lysis buffer (ELB) (120 mM sodium chloride, 50 mM Tris/HCl, pH 7.4, 20 mM sodium fluoride, 1 mM EDTA, 6 mM EGTA, 15 mM sodium pyrophosphate, 1 mM PMSF, 0.5% Nonidet P-40, 1 ng/ml of aprotinin, 0.2 ng/ml of leupeptin, 1 ng/ml of pepstatin) and centrifuged for 20 min at 14 000 × *g*. 10% of the supernatant was retained for input control, the remaining 90% was incubated with GFP-trap beads (Chromotec, München) for 3 h at 4°C. Beads were washed three times with ELB buffer, boiled in sodium dodecyl sulfate (SDS) sample buffer and analyzed together with the input samples by SDS-(PAGE) polyacrylamide gel electrophoresis and western blotting. For co-immunoprecipitation, the desired antibodies were added to the total cell extract (TCE) and incubated for 2 h at 4°C. Immune complexes were then bound to protein A sepharose, washed and analyzed by western blotting.

### GST pull-down assay

Glutathione S-transferase (GST) fusion protein expression was induced in cultures of transformed *Escherichia coli* BL21-pLysS bacteria by adding isopropyl-d-thiogalactopyranoside to a final concentration of 0.5 mM. After additional 3 h of growth at 37°C the bacteria were harvested by centrifugation for 10 min at 5000 × *g*. Bacterial pellets were resuspended in GST lysis buffer (50 mM Tris-HCl; pH 8,0; 150 mM NaCl; 1% Triton X-100; 1 mM DTT; 0,1 mM Phenylmethylsulfonyl fluoride (PMSF)) and lysed by three freeze-thaw-cycles and sonication. An extract of soluble protein was prepared by ultracentrifugation for 1 h at 100 000 × *g*. Extracts containing 5–10 μg of GST fusion protein were then mixed with 30 μl of glutathione-sepharose (GE Healthcare) and incubated at 4°C for 1 h. The sepharose beads were then washed three times with ELB buffer and used for GST pull-down assays as follows: QT6 cells transfected with the appropriate expression vectors were lysed in ELB buffer and aliquots of the lysate were then incubated under constant agitation for 1 h at 4°C with bacterially expressed GST fusion protein coupled to glutathione-sepharose. Alternatively, GST fusion protein-loaded sepharose beads were incubated with purified, bacterially expressed His-PABP-RRM1–4. Subsequently, beads were washed three times with ELB buffer. Bound proteins were eluted from the beads by boiling in SDS sample buffer and analyzed by SDS-PAGE followed by staining with Coomassie brilliant blue or western blotting using appropriate antibodies.

### Sucrose density gradient centrifugation of cytoplasmic extracts

Cells were treated with 50 μg/ml cycloheximide for 30 min before they were washed with ice-cold PBS (containing 50 μg/ml cycloheximide) and lysed for 20 min on ice in hypotonic buffer (10 mM Hepes, pH 7.5; 5 mM KCl; 2 mM MgCl_2_; 1 mM DTT; 0.5% NP40; 1 mM PMSF; supplemented with 40 U/ml RNaseOUT (Invitrogen) and a protease inhibitor cocktail containing 5 ng/ml pepstatin A, 1 ng/ml leupeptin hemisulfate and 5 ng/ml aprotinin). The lysed cells were pelleted at 14 000 rpm for 15 min at 4°C. The supernatant was collected as cytoplasmic fraction and layered on top of a 10–50% sucrose density gradient made in hypotonic buffer. Gradients were centrifuged in a SW41 rotor at 36 000 rpm for 2 h at 4°C. The gradients were fractionated and aliquots of the fractions were analyzed by 10% SDS-PAGE and western blotting to determine the distribution of Pdcd4, or treated with Proteinase K and 1% SDS and analyzed by agarose gel electrophoresis to determine the distribution of ribosomal RNAs.

### RNA-binding assays

RNA-binding assays of GST-Pdcd4 fusion proteins were performed as described before in (16). GST fusion proteins were fractionated by SDS-PAGE and blotted onto nitrocellulose. Radiolabeled RNA was synthesized *in vitro* using T7 RNA polymerase, [α^32^P]-UTP and the linearized plasmid pCDNA3 c-myb#3 (27) as template. Blots were incubated first with 6 M urea; 0.2% NP40 for 10 min, washed four times 15 min each with RNA-binding buffer (10 mM Tris HCl, pH 7.5; 5 mM KCl; 2 mM MgCl_2_) lacking RNA and then incubated for 15 min in RNA-binding buffer containing radiolabeled RNA. Subsequently, the blots were washed three times for 10 min with binding buffer lacking RNA and analyzed by autoradiography. All incubations were performed at room temperature.

Chromatography on poly(U)-agarose was performed as follows (all steps at 4°C). GST-Pdcd4 fusion proteins were purified by binding to glutathione-sepharose and eluting them over night with 15 mM glutathione in 10 mM Tris-HCl, pH 7.5. Eluted proteins were then incubated for 1–2 h with poly(U)-agarose (Sigma-Aldrich) equilibrated in binding buffer (10 mM Hepes, pH 7.5; 5 mM KCl; 5 mM EDTA). The agarose beads were then transferred to a chromatography column, washed several times with binding buffer to remove unbound protein. Bound proteins were then eluted with binding buffer supplemented with increasing concentrations of NaCl. Aliquots of the input sample, the last wash fraction and the salt elution fractions were then analyzed by SDS-PAGE and western blotting.

### Electrophoretic mobility shift assay

Electrophoretic mobility shift assay (EMSA) was performed with an oligo(A)_25_ ribo-oligonucleotide end-labeled with [γ^32^P]-ATP and T4 polynucleotide kinase. His-PABP-RRM1–4 was purified from bacterial extracts by binding to Ni-NTA beads followed by washing with purification buffer (50 mM NaH_2_PO_4_, pH 8.0; 5 mM NaCl) and eluting the proteins in the same buffer supplemented with 200 mM imidazole as described above. Binding reactions were set up in a total volume of 20 ml of buffer (10 mM Tris-HCl, pH 7.8; 100 mM NaCl, 1 mM EDTA, 2% ficoll) containing the radiolabeled oligo(A)_25_ and the desired proteins. Reactions were incubated for 30 min on ice and analyzed by electrophoresis in 7.5% native polyacrylamide gels in 0.5 × TBE buffer. Gels were dried and analyzed with a phosphor image analyzer.

## RESULTS

### Pdcd4 interacts with PABP *in vitro* and *in vivo*

**Figure 1.**
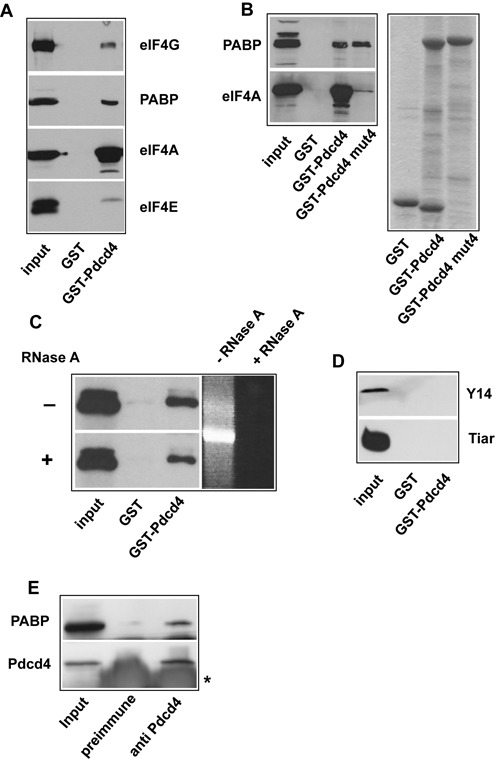
Pdcd4 interacts with PABP. (**A** and **B**) GST, GST-Pdcd4 or GST-Pdcd4 mut4 proteins were immobilized on glutathione sepharose and incubated with HEK293 cell extract. Bound proteins and aliquots of the total extracts were analyzed by SDS-PAGE and western blotting with antibodies against eIF4A, eIF4E, eIF4G and PABP. A Coomassie blue stained gel demonstrating equal loading of the beads with GST proteins is shown on the right side of (B). (**C**) GST pull-down experiment showing the binding of PABP from extract of HEK293 cells. Binding reaction was performed in the absence or presence of 0.5 μg/ml RNase A. The right side shows an agarose gel of *in vitro* translated RNA incubated without or with RNase A under the same conditions. (**D**) Extract of HEK293 cells (top) or of cells transfected with a Flag-Tiar expression vector (bottom) was analyzed for binding to GST and GST-Pdcd4 as in (A). Proteins were detected with antibodies against Y14 and the Flag-tag. (**E**) Extract from HEK293 cells was precipitated with rabbit antiserum against Pdcd4 or with pre-immune serum. Immunoprecipitates and an aliquot of the TCE (input) were analyzed by western blotting with antibodies against PABP and Pdcd4. The asterisk marks an intense signal in the immunoprecipitate lanes, which is due to immunoglobulins from the antiserum.

We have shown previously that Pdcd4 suppresses the translation of c-*myb* and A-*myb* mRNAs by binding to RNA secondary structures present in the coding regions of these mRNAs (27,28). Unexpectedly, the inhibitory effect exerted by Pdcd4 on these RNAs was also observed when a mutant of Pdcd4 was used that was not able to interact with the translation initiation factor eIF4A (28; and data not shown). This mutant (Pdcd4-mut4) contained several amino acid substitutions in both MA3 domains that were previously shown to be essential for eIF4A binding (18–24). Therefore, we became interested to investigate whether Pdcd4 interacts also with other translation factors in addition to eIF4A. *In vitro* binding experiments using GST-Pdcd4 and extracts of human cells showed that Pdcd4 was able to bind eIF4A, eIF4G, eIF4E and PABP present in the extract (Figure 1A). Comparison of the input and bound fractions of these proteins suggested that eIF4A and PABP were bound more efficiently than eIF4G and eIF4E. We therefore focused on the possibility that Pdcd4 also binds to PABP in addition to its known interaction partner eIF4A. To investigate whether the binding of PABP by GST-Pdcd4 was mediated by eIF4A, we performed a pull-down experiment with a Pdcd4 mutant (Pdcd4-mut4) that is unable to interact with eIF4A (18–24). This experiment clearly showed that the binding of PABP to Pdcd4 was independent of the Pdcd4-eIF4A interaction and suggested that PABP might be a direct interaction partner of Pdcd4 (Figure 1B). GST-Pdcd4-mut4 also showed reduced binding of eIF4G and eIF4E suggesting that their interaction with Pdcd4 was mediated by eIF4A (data not shown). Since PABP and Pdcd4 are RNA-binding proteins, we were concerned that the binding of PABP to GST-Pdcd4 might be due to RNA present in the cell extract. To address this possibility we performed an additional pull-down experiment to see if RNase A added during the binding reaction diminished the binding (left part of Figure 1C). A control reaction containing *in vitro* transcribed RNA and incubated under the same conditions confirmed that the amount of RNase A added to the binding reaction was sufficient to completely digest the RNA (right part of Figure 1C). Moreover, we also analyzed the binding of two unrelated RNA-binding proteins to GST-Pdcd4. Figure 1C shows that the RNA-binding protein Tiar and the exon-junction complex protein Y14 were not bound by Pdcd4, pointing toward a direct interaction between Pdcd4 and PABP. Together, these experiments made it unlikely that the interaction of Pdcd4 and PABP was mediated by unspecific binding to RNA present in the cell extract. To provide evidence that Pdcd4 and PABP also interact as endogenous proteins, we performed co-immunoprecipitation experiments with extracts from untransfected HEK293 cells. Figure 1D shows that antiserum against Pdcd4, but not the pre-immune serum used as control, was able to coprecipitate a significant fraction of PABP from cell extracts.

### The interaction of Pdcd4 and PABP is mediated by the RNA-binding domains of both proteins

**Figure 2.**
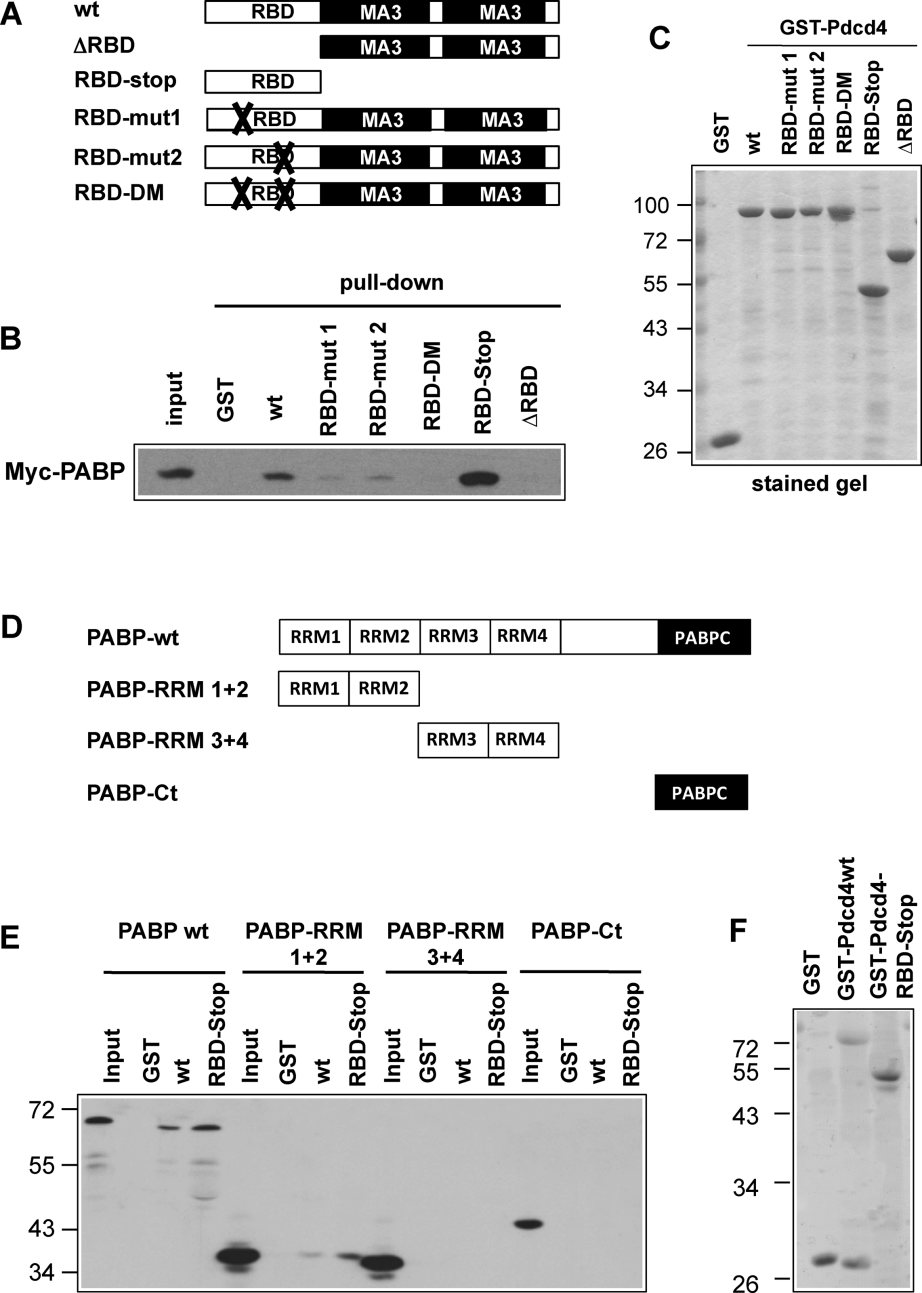
Mapping of the Pdcd4- and PABP-binding regions. (**A**) Schematic structure of GST-Pdcd4 proteins. RBD and MA3 refer to the RNA binding and MA3 domains. (**B**) Cell extract from QT6 fibroblasts transfected with an expression vector for Myc-PABP was analyzed for binding to GST-Pdcd4 proteins as in Figure 1. Bound proteins and an aliquot of the input extract were analyzed by western blotting with antibodies against the Myc-tag. (**C**) Coomassie blue stained gel demonstrating equal loading of the sepharose beads with GST-Pdcd4 proteins. (**D**) Schematic structure of Myc-PABP proteins. RRM1–4 and PABPC refer to the RNA-binding motifs and the C-terminal domain of PABP. (**E**) Cell extract from QT6 fibroblasts transfected with an expression vectors for Myc-PABP constructs was analyzed for binding to GST-Pdcd4 proteins. Bound proteins and an aliquot of the input extracts were analyzed by western blotting with antibodies against the Myc-tag. (**F**) Equal loading of the sepharose beads with GST-Pdcd4 proteins is shown by Coomassie blue staining.

To map the binding site for PABP within Pdcd4, we performed *in vitro* binding experiments using wild-type and the mutant GST-Pdcd4 proteins illustrated schematically in Figure 2A. The purified proteins are shown in Figure 2C. Figure 2B shows that a Pdcd4 deletion construct consisting only of the N-terminal RNA-binding domain was able to bind PABP, whereas a deletion mutant lacking the RNA-binding domain but containing both MA3 domains failed to bind PABP. This showed that PABP interacts with the RNA-binding domain of Pdcd4 and confirmed that binding of eIF4A and PABP are independent binding partners of Pdcd4. We also tested the binding of PABP to Pdcd4 proteins carrying amino acid replacements in one or both of two clusters of basic amino acids (referred to as RBD mut1, RBD mut2 and RBD-DM) that were found previously to be crucial for RNA binding by Pdcd4 (25). Both single RBD mutants showed reduced binding of PABP and the double mutant did not bind to PABP at all (Figure 2B). This substantiated the conclusion that PABP interacts with the RNA-binding domain of Pdcd4.

To map the binding site for Pdcd4 within PABP, we performed *in vitro* pull-down experiments using partially deleted versions of PABP (Figure 2D). As shown in Figure 2E and F, only full-length PABP and the construct consisting of RRM motifs 1 and 2 was able to bind to Pdcd4. We therefore concluded that the main interaction site for Pdcd4 is located within RRM 1 and 2 of PABP. However, because the efficiency of binding of full-length PABP to GST-Pdcd4 was higher than that of PABP-RRM1 + 2 it is possible that another part of PABP also contributes to the interaction with Pdcd4.

### Pdcd4 and PABP are direct interaction partners

**Figure 3.**
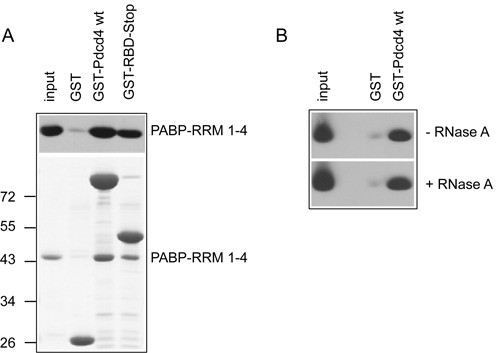
PABP and Pdcd4 are direct interaction partners. (**A**) Bacterially expressed and purified PABP-RRM 1–4 was analyzed for binding to GST-Pdcd4. Input and bound proteins were analyzed by western blotting with antibodies against PABP (top) or by staining with Coomassie blue (bottom). The input fraction corresponds to 2.5% of the bound fractions. (**B**) Binding of PABP-RRM 1–4 to the indicated GST-proteins was carried out in the absence or presence of 0.5 μg/ml RNase A, followed by western blotting with antibodies against PABP.

Because PABP was derived from extracts of transfected cells in the binding experiments shown in Figure 2, we could not exclude the possibility that the interaction of Pdcd4 and PABP was mediated by another protein present in the cell extract. We therefore expressed His-PABP-RRM1–4 in bacteria, purified it and subjected it to a pull-down experiment with bacterially expressed and purified GST-Pdcd4. Figure 3 shows that bacterially expressed PABP-RRM1–4 bound efficiently to GST-Pdcd4 and to a GST-Pdcd4 protein containing only the RNA-binding domain of Pdcd4. Since both purified proteins interacted in the absence of other eukaryotic proteins, we concluded that Pdcd4 and PABP are directly binding to each other. We also confirmed by a binding reaction in the presence of RNase A that the interaction was not mediated by unspecific binding of both proteins to RNA (Figure 3B).

### The ability to bind to RNA and to interact with PABP is conserved between human and Drosophila Pdcd4

**Figure 4.**
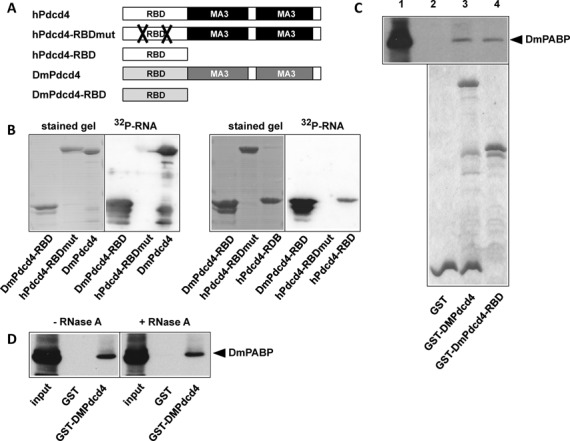
The ability of Pdcd4 to bind to RNA and PABP is conserved between humans and *Drosophila melanogaster*. (**A**) Schematic structure of human and Drosophila Pdcd4. (**B**) Bacterially expressed and purified human and Drosophila GST-Pdcd4 proteins were fractionated by SDS-PAGE and stained with Coomassie blue or blotted onto nitrocellulose. The blots were incubated with radiolabeled RNA, washed and analyzed by autoradiography. (**C**) Extract from Drosophila Schneider S2 cells was incubated with the indicated GST proteins immobilized on glutathione sepharose beads. Bound proteins and an aliquot of the TCE was analyzed by western blotting with antibodies against Drosophila PABP (top). A stained gel demonstrating equal loading of the beads with GST proteins is shown below. (**D**) Extracts from Drosophila Schneider S2 cells were analyzed for binding to the indicated GST proteins as in (C). Binding reactions were performed in the absence or presence of 0.5 μg/ml RNase A.

Evolutionary conservation provides strong arguments for the functional importance of specific traits of proteins, such as their ability to undergo specific interactions. Since Pdcd4 has been highly conserved during evolution, we were interested to know whether the ability to bind to RNA and to interact with PABP are conserved features of Pdcd4. The *CG10990* gene of *Drosophila melanogaster* encodes a protein that is highly related to the mammalian Pdcd4 protein. Figure 4A shows that the domain structures of human and Drosophila Pdcd4 are very similar. To investigate if the Drosophila protein has RNA-binding activity, we employed a nitrocellulose blotting assay. Purified bacterially expressed human and Drosophila GST-Pdcd4 proteins were subjected to SDS-PAGE and blotted onto a nitrocellulose membrane or stained with Coomassie blue. The blots were then incubated with radiolabeled *in vitro* synthesized RNA, washed and analyzed by autoradiography. Figure 4B shows that human and Drosophila Pdcd4 were able to bind RNA. As a control for specificity of the binding assay a mutant version of human Pdcd4 (Pdcd4-RBDmut) lacking RNA-binding activity (25) was included. The experiment also showed that the N-terminal domain of the Drosophila protein, like that of human Pdcd4 (16,25), is sufficient for RNA binding. To investigate whether Drosophila Pdcd4 also interacts with PABP we performed an *in vitro* binding experiment with TCE from Drosophila Schneider S2 cells. Figure 4C shows that full-length Drosophila Pdcd4 as well as the N-terminal domain of the protein were able to bind PABP. Binding also occurred when RNase A was added to the cell extract, suggesting that it was not an artifact of unspecific binding of both proteins to RNA (Figure 4D). We therefore concluded that the N-terminal domain of Drosophila Pdcd4 binds RNA as well as PABP, demonstrating that both binding activities are conserved during evolution.

### Mutation of highly conserved amino acid residues within the Pdcd4 RNA-binding domain abolishes its interaction with PABP

**Figure 5.**
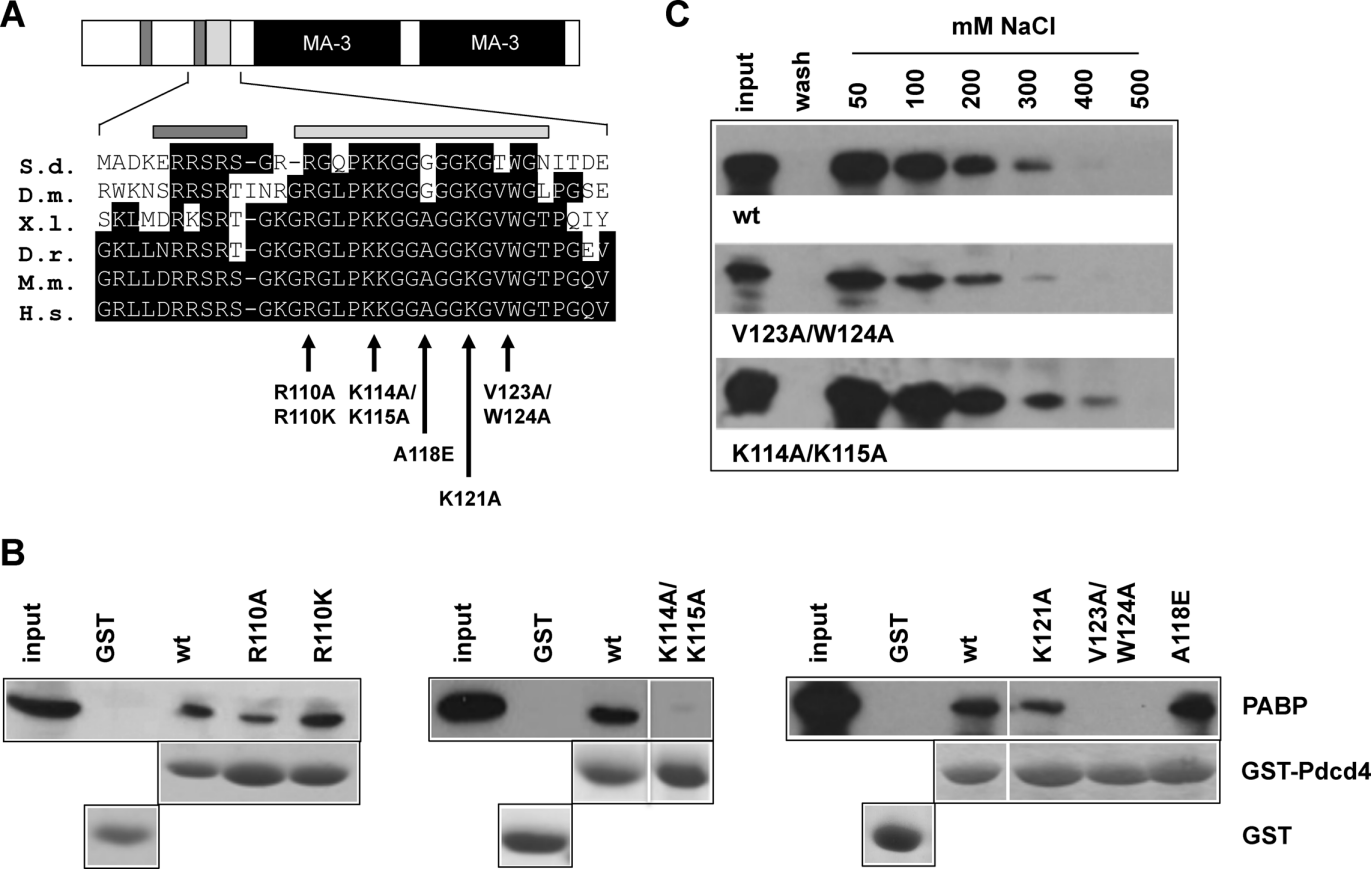
PABP- and RNA-binding are independent activities mediated by the N-terminal domain of Pdcd4. (**A**) Amino acid sequences of part of the N-terminal domain of Pdcd4 from different species. S.d., *Suberites domuncula*; D.m., *Drosophila melanogaster*; X.l., *Xenopus laevis*; D.r., *Danio rerio*; M.m., *Mus musculus*; H.s., *Homo sapiens*. Clusters of basic amino acids involved in RNA binding are marked by yellow boxes. Light and dark grey boxes mark the regions of highest conservation within the N-terminal domain and the clusters of basic amino acids implicated in RNA binding, respectively. (**B**) Extracts from cells expressing Myc-PABP were analyzed for binding to wild type and mutant GST-Pdcd4 proteins. Input and bound Myc-PABP was detected by western blotting with anti-Myc antibodies. GST-proteins were detected by Coomassie blue staining. Only the bands corresponding to the respective GST proteins are shown. (**C**) GST-Pdcd4-wt, GST-Pdcd4-V123A/W124A and GST-Pdcd4-K114A/K115A were analyzed by chromatography on poly(U)-agarose. Bound proteins were eluted from the column with binding buffer containing increasing amounts of NaCl. Samples of input, wash and salt-eluate fractions were analyzed by western blotting with Pdcd4 antiserum.

To further explore the role of the interaction of Pdcd4 with PABP, we decided to generate Pdcd4 mutants that disrupt the PABP-binding activity. Comparison of the Pdcd4 amino acid sequence of widely divergent species revealed a very highly conserved region within the second half of the N-terminal domain that has not been implicated in RNA binding (Figure 5A). To investigate if this region is involved in PABP binding, we mutated specific amino acid residues in this conserved region. These mutants were expressed as GST-Pdcd4 fusion proteins and examined in pull-down experiments for their ability to interact with PABP. As shown in Figure 5B, two of the mutants (K114A/K115A and V123A/W124A) were unable to interact with PABP, demonstrating that the highly conserved part of the RNA-binding domain is involved in the Pdcd4-PABP interaction. We also analyzed the RNA-binding activity of these mutants by a poly(U)-agarose-binding assay (Figure 5C). Wild-type Pdcd4 and both mutant proteins were able to bind to poly(U)-agarose and were eluted at similar salt concentrations, demonstrating that both Pdcd4 mutants had retained RNA-binding activity. Thus, the Pdcd4 mutants K114A/K115A and V123A/W124A dissociate RNA- and PABP-binding activities of Pdcd4 from each other, clearly demonstrating that these are independent activities of Pdcd4.

### Pdcd4 mutants lacking PABP-binding activity do not suppress translation of c-*myb* mRNA

**Figure 6.**
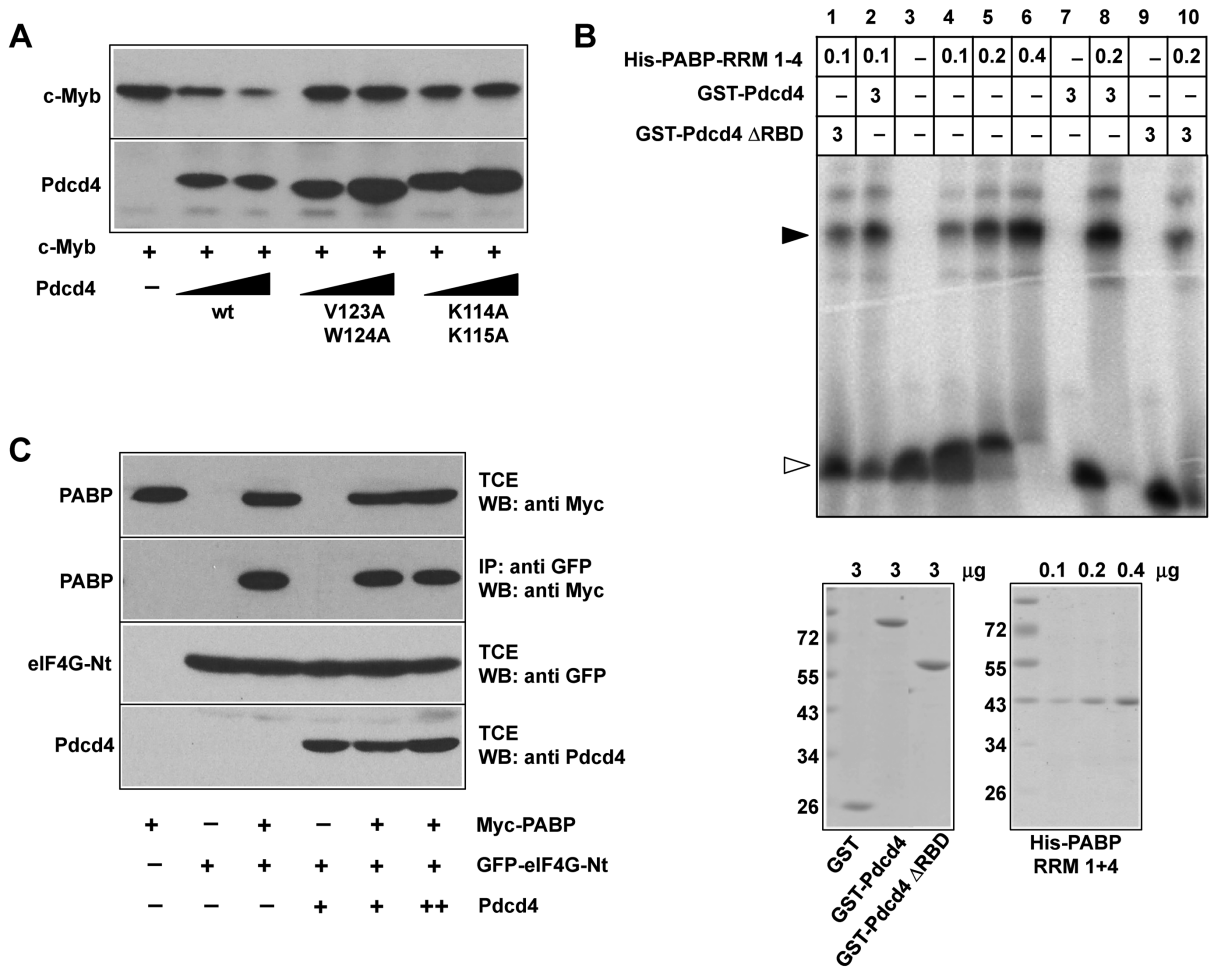
Pdcd4 mutants lacking PABP-binding activity do not suppress translation of c-*myb* mRNA. (**A**) QT6 fibroblasts were transfected with expression vectors for c-Myb and wild-type or mutant Pdcd4, as indicated. TCEs were analyzed by western blotting with antisera against c-Myb and Pdcd4. (**B**) Bacterially expressed and purified His-PABP RRM1–4, GST-Pdcd4 and GST-Pdcd4 ΔRBD were subjected to electrophoretic mobility shift assays using a radiolabeled oligo(A)_25_ ribo-oligonucleotide. Oligonucleotides were incubated with the indicated amounts (in μg) of the proteins and analyzed by electrophoresis in a nondenaturing polyacrylamide gel. The black and white arrowheads mark RNA-protein complexes and the free oligonucleotide, respectively. Coomassie-stained SDS-polyacrylamide gels demonstrating the purity of the proteins are shown below. (**C**) QT6 fibroblasts were transfected with expression vectors for Myc-PABP, Pdcd4 and a GFP-eIF4G fusion protein encompassing the binding site for PABP. TCEs were analyzed by western blotting with the indicated antibodies. Cell extracts were also subjected to immunoprecipitation with antibodies against GFP, followed by western blotting of the immunoprecipitates with Myc-specific antibodies.

To explore the functional relevance of the interaction of Pdcd4 and PABP, we first investigated if the Pdcd4 mutants that failed to bind to PABP were still able to suppress the translation of c-*myb* mRNA. Figure 6A shows that wild-type Pdcd4 suppressed the expression of c-Myb when co-transfected with a c-Myb expression vector, consistent with our previous work which has shown that the inhibitory effect of Pdcd4 is due to suppression of translation of c-*myb* RNA (27). Interestingly, both Pdcd4 mutants were unable to reduce c-Myb expression, suggesting that the interaction with PABP is necessary for the suppression of c-*myb* mRNA translation by Pdcd4.

PABP is known to stimulate translation initiation by interacting with eIF4G bound at the 5′ end of the mRNA, thereby causing formation of a closed loop structure of the mRNA (36,37). Several proteins have been shown to suppress translation by interfering with the circularization of the RNA. For example, the interaction with the PABP-interacting protein 2 (Paip2) displaces PABP from the poly(A)-tail of the mRNA and also inhibits its interaction with eIF4G (38,39). Similarly, the GW182 protein, which acts as a scaffold for the assembly of silencing complexes on specific mRNAs, is thought to disrupt PABP function also by blocking its interaction with eIF4G (40). To see if Pdcd4 acts in similar manner, we investigated the effect of Pdcd4 on the ability of PABP to bind to the poly(A)-tail and to interact with eIF4G. To study the influence of Pdcd4 on RNA binding by PABP, we performed EMSAs with a radiolabeled oligo(A)25 ribo-oligonucleotide and bacterially expressed, purified PABP-RRM1–4, GST-Pdcd4 and GST-Pdcd4-ΔRBD. Figure 6B shows that PABP bound strongly to the oligonucleotide, as evidenced by the mobility shift of the oligonucleotide, whereas Pdcd4 itself did not bind. Addition of an excess of Pdcd4 to the binding reaction containing PABP did not reduce the PABP-induced mobility shift. We therefore concluded that Pdcd4 does not interfere with RNA-binding activity of PABP.

To address if Pdcd4 interferes with the interaction between PABP and eIF4G, we transfected fibroblasts with different combinations of expression vectors for Pdcd4, Myc-PABP and a GFP-eIF4G fusion protein that contains the binding site for PABP. TCEs were then analyzed by immunoprecipitation using sepharose beads carrying a covalently bound high-affinity GFP-binding protein. As expected, PABP was specifically precipitated via eIF4G, however, co-expression of Pdcd4 had no effect on this interaction (Figure 6C). Taken together, these experiment suggested that Pdcd4 does not suppress translation by interfering with the PABP-eIF4G interaction or with PABP RNA binding.

### Pdcd4 mutants lacking PABP-binding activity do not cosediment with ribosomal complexes

**Figure 7.**
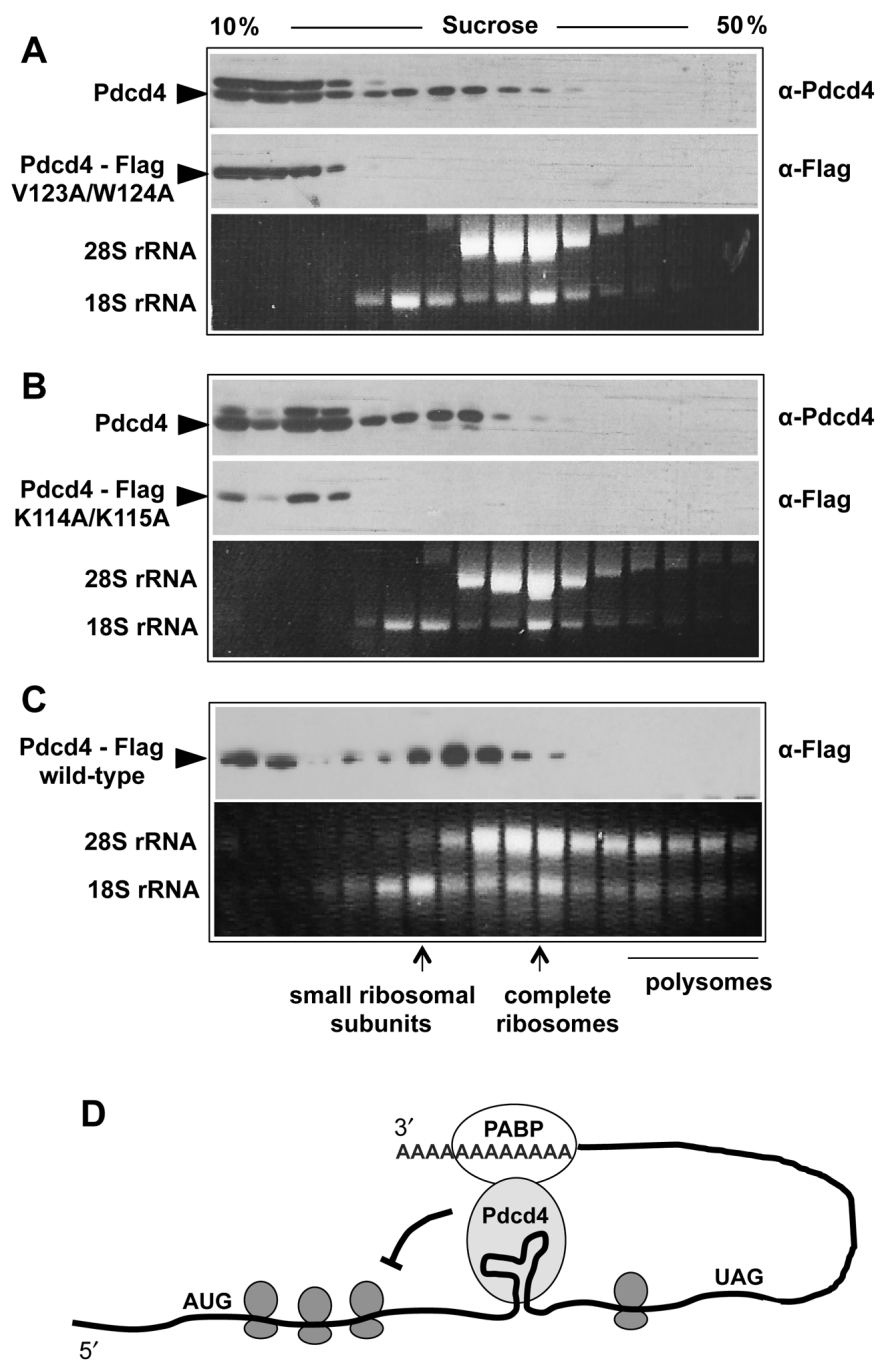
Ribosomal association of mutant Pdcd4 proteins. Hela cells were transfected with expression vectors for Flag-tagged Pdcd4-V123A/W124A (**A**), Pdcd4-K114A/K115A (**B**) or Flag-tagged wild-type Pdcd4 (**C**). Cytoplasmic extracts were prepared 20 h after transfection and fractionated by sucrose density gradient sedimentation. Individual gradient fractions were analyzed by western blotting with antibodies against Pdcd4 (top), the Flag-tag (middle) and by agarose gel electrophoresis to detect 18S and 28S ribosomal RNAs (bottom). In (C), western blotting were performed only with anti-Flag antibodies. The arrowheads mark endogenous and mutant Pdcd4. Because of the Flag-tag the mutant proteins have a higher molecular weight than endogenous Pdcd4 and are also visible in the top panels. (**D**) Tentative model of the regulation of c-*myb* mRNA translation by Pdcd4. Pdcd4 binds to a secondary structure formed by a part of the coding region of c-*myb* mRNA and inhibits translational elongation. The association of Pdcd4 with the RNA is stabilized by interaction with PABP.

Fractionation of cytoplasmic extracts by sucrose density gradient centrifugation has previously shown that Pdcd4 is associated with eIF4A-containing ribosomal complexes. These complexes sediment slightly faster than small ribosomal subunits but slower than complete ribosomes and are thought to be translation pre-initiation complexes (25). To investigate if the disruption of the Pdcd4-PABP interaction affects the association of Pdcd4 with these complexes, we transfected expression vectors for the Flag-tagged Pdcd4-K114A/K115A and V123A/W124A mutants into Hela cells and fractionated cytoplasmic extracts of these cells in sucrose density gradients. Analysis of the gradient fractions showed that both mutant proteins had lost the ability to associate with ribosomal complexes and appeared only in the upper part of the gradient (Figure 7A and B). A control experiment using Flag-tagged wild-type Pdcd4 confirmed that the Flag-tag by itself did not prevent interaction with ribosomal complexes (Figure 7C). Overall, these experiments indicated that the interaction with PABP is required for the association of Pdcd4 with the ribosomal complexes.

## DISCUSSION

The tumor suppressor gene *Pdcd4* is attracting increasing attention because its expression is frequently downregulated in different types of human tumors. In many cases downregulation appears to be due to increased expression of micro-RNA miR-21, an onco-miR that is upregulated in many cancer cells (41). The function of Pdcd4 is less well understood, particularly with respect to its role as a translation suppressor. The finding that Pdcd4 inhibits the helicase activity of translation initiation factor eIF4A has initially suggested that Pdcd4 acts as a translation suppressor of mRNAs with structured 5′ UTRs. That Pdcd4 can suppress the translation of such RNAs has indeed been confirmed by analysis of the effect of Pdcd4 on artificial RNA constructs that form stable stem-loop structures at their 5′ ends (18,33). However, since natural Pdcd4 target mRNAs were initially not known, several important questions have remained open: which mRNAs are regulated by Pdcd4? Does Pdcd4 suppress the translation of all mRNAs containing structured 5′ UTRs? Are there alternative mechanisms of translational suppression by Pdcd4? Are there mechanisms that allow Pdcd4 to selectively target specific mRNAs?

We have recently identified several natural Pdcd4 target RNAs, which has allowed us to address some of these questions and to investigate the mechanism of translational suppression by Pdcd4 in more detail (14,27,28). By examining the effect of Pdcd4 on p53 mRNA, a natural mRNA with a highly structured 5′ UTR, we have confirmed the proposed mechanism of translational suppression by Pdcd4. We have shown that the inhibition of p53 mRNA translation by Pdcd4 is mediated by the 5′ UTR and depends on the ability of Pdcd4 to bind to eIF4A (14). Surprisingly, the analysis of the effect of Pdcd4 on c-*myb* and A-*myb* mRNAs has indicated that there is an additional mechanism of translational suppression by Pdcd4, which is independent of the Pdcd4/eIF4A-interaction, but instead requires the RNA-binding activity of Pdcd4 to target Pdcd4 to these mRNAs. We have shown that Pdcd4 preferentially binds to RNA secondary structures formed by the coding regions of c-*myb* and A-*myb* mRNAs (27,28). As a clear indication that these binding regions indeed mediate the specific targeting of Pdcd4 to these RNAs, we have shown that the fusion of the secondary structure regions to a heterologous RNA allowed Pdcd4 to recognize these RNAs as targets for translational suppression. In the course of these experiments, we have observed that translation suppression by Pdcd4 was abolished when a translation stop codon was introduced into the mRNA upstream of the Pdcd4-binding region (28). This demonstrated that Pdcd4 mediates translation suppression of these mRNAs only when it binds to the translated part of the RNAs, which suggested that Pdcd4 suppresses the translation of these RNAs by inhibiting translation elongation. Taken together, these analyses have shown that there are at least two distinct mechanisms by which Pdcd4 supresses translation, namely inhibition of translation initiation via binding to eIF4A, and inhibition of translation elongation by direct binding of Pdcd4 to the coding region of specific RNAs.

The identification of PABP as a Pdcd4 interaction partner extends our picture of Pdcd4′s role as a translation suppressor and shows that the cooperation of Pdcd4 with the translational machinery is more complex than previously known. Our mapping experiments have shown that Pdcd4 and PABP interact by direct protein–protein interactions via their RNA-binding domains. The part of Pdcd4 implicated by our mutation analysis in the interaction with PABP shows the highest evolutionary conservation within the N-terminal domain of the protein, underling the importance of this region for the function of Pdcd4. Indeed, the ability of Pdcd4 to bind to RNA and to interact with PABP have been highly conserved during evolution, as demonstrated by the analysis of the Drosophila homolog of Pdcd4.

PABP is an important component of the translation machinery but also plays key roles in other processes, such as the regulation of mRNA stability. PABP enhances the translation of capped and polyadenylated RNAs by interacting with eIF4G and forming a closed loop structure of the mRNA (36,37). PABP also affects the formation of the translation initiation complex at subsequent steps, such as the joining of the ribosomal subunits (42–44). A variety of protein–protein interactions involving the RNA-binding and C-terminal domains of PABP have been shown to modulate the function of PABP in translation initiation, mRNA stability and micro-RNAs mediated silencing (40,45–47). How does the interaction with Pdcd4 fit into this picture? Our *in vitro* RNA-binding experiments have shown that Pdcd4 does not disrupt the binding of PABP to the poly(A) tail or interfere with the interaction of PABP with eIF4G. The role of Pdcd4 is therefore clearly different from that of the Paip2, which also binds to the RNA-binding domain of PABP but suppresses translation by disrupting PABP RNA binding and its interaction with eIF4G (38,39). We have shown that Pdcd4 mutants that are defective for binding to PABP have lost the ability to stably associate with ribosomal complexes during sucrose density sedimentation. This suggests that the Pdcd4-PABP interaction is required for tethering Pdcd4 to these complexes. Consistent with the notion that the interaction with PABP stabilizes Pdcd4 on its mRNA targets our data show that Pdcd4 mutants that do not interact with PABP, fail to suppress the translation of c-*myb* mRNA. If Pdcd4 in this case blocks translation elongation by directly binding to sequences located within the coding region of the RNA, as suggested by our previous work, one might suspect that translating ribosomes will weaken or disrupt the Pdcd4-RNA interaction. The ability of Pdcd4 to bind PABP might therefore be important to stabilize Pdcd4 on the RNA or allow it to rebind to the RNA in case it is displaced by an approaching ribosome. Thus, PABP appears to function as a cofactor helping Pdcd4 to exert its suppressive function. Figure 7D depicts a tentative model for the regulation of c-*myb* mRNA translation by Pdcd4. Some aspects of this model are reminiscent of the translation inhibition of Drosophila *msl-2* mRNA by the SXL-UNR co-repressor complex (44). The SXL-UNR complex binds PABP without interfering with the PABP-eIF4G interaction and closed-loop formation of the *msl-2* RNA, suggesting that the SXL-UNR complex requires PABP as a cofactor for translation suppression. It is worth pointing out that the Pdcd4 RNA-binding domain is the target of post-translational modifications, such as phosphorylation of serine 67 by p70s6K or AKT kinases (17,48) or methylation of arginine 110 by PRMT5 (26). It is conceivable that these modifications also affect the binding of PABP, thereby linking translational suppression of specific mRNAs by Pdcd4 to signal transduction pathways.

In summary, our work demonstrates that Pdcd4 interacts with the PABP, a key player in the control of translation. This interaction has been highly conserved during evolution and contributes to the role of Pdcd4 as translation suppressor. Given the multifunctional role of PABP in translation and RNA-metabolism our work also raises the possibility that Pdcd4, by modulating the binding or function of other PABP-interacting proteins, might also affect other functions of PABP.
